# Early Orthopaedic Treatment of Hemifacial Microsomia

**DOI:** 10.1155/2017/7318715

**Published:** 2017-12-14

**Authors:** Diana Cassi, Marisabel Magnifico, Mauro Gandolfinini, Ilda Kasa, Giovanni Mauro, Alberto Di Blasio

**Affiliations:** Section of Orthodontics, Centro Universitario di Odontoiatria, Department of Medicine and Surgery, University of Parma, Parma, Italy

## Abstract

The aim of this paper is to report treatment effects of functional therapy in a growing patient affected by hemifacial microsomia (HM). According to Kaban's classification, the patient was classified as grade IIa as she presented all mandibular and temporomandibular joint components and a normal shaped, hypoplastic mandible. The therapeutic approach included the use of an asymmetrical functional activator (AFA) to stimulate the growth of the affected side and consequently to improve symmetry of the mandible and maxillary deficiency. Further effects were the lengthening of the mandibular ramus, restoration of occlusion, and expansion of soft tissues.

## 1. Introduction

Hemifacial microsomia (HM) is a congenital craniofacial malformation caused by hypoplasia of anatomical structures deriving from the first and second branchial arches. As a result, HM involves facial skeleton, soft tissues, ear, and cranial nerves, thus resulting in the absence or insufficiency of these components [1]. Such a congenital malformation is also known as Goldenhar syndrome, craniofacial microsomia, first and second branchial arch syndrome, otomandibular dysostosis, and lateral facial dysplasia [2].

HM is the second most frequent craniofacial birth defect after cleft lip and palate. Reported incidence varies from 1 case every 3000 to 1 case every 5600 newborns [3].

Males and females are not equally affected: females are less frequently affected than males with an estimated ratio of 2 : 3. There is also a difference between the affected sides: a right malformation is more frequent than the left one (ratio: 3 : 2) [4].

Aetiology of this condition is uncertain although the majority of authors suggest the hypothesis of an early hemorrhage from the stapedial artery causing a hematoma in the supplied region. Thus, different phenotypical grades of dysmorphism may occur according to the degree of vascular damage.

Among various classification systems proposed, in the present article, we selected and used the classification proposed by Kaban [5], which distinguishes the following categories according to the severity of the anatomical anomalies:

  Type I: hypoplastic temporomandibular joint
  Type IIa: hypoplastic and abnormal shape of mandibular ramus, condyle, and temporomandibular joint
  Type IIb: mandibular ramus is hypoplastic and markedly abnormal in form and location, being medial and anterior
  Type III: absence of mandibular ramus
  Type IV: mandibular body hypoplasia

Treatment of patients depends on the degree of deformity of the facial structures and includes repair of bony asymmetry as well as of soft tissue defects and auricular anomalies [6].

The purpose of this report was to present a nonsurgical approach to treat a young girl affected by mild type IIa HM, using an asymmetrical functional activator (AFA).

## 2. Case Report

A 2-year-old girl was referred to the Department of Oral and Maxillofacial Surgery and Department of Orthodontics of Parma University, Italy, for the evaluation and treatment of a facial deformity.

**Figure 1:**
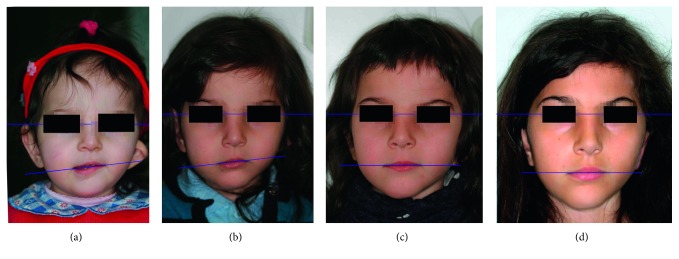
Frontal photographs showing the progressive levelling of oral commissure during treatment and follow-up: (a) 2 years and 1 month; (b) 3 years and 11 months; (c) 4 years and 10 months; (d) 7 years and 8 months.

**Figure 2:**
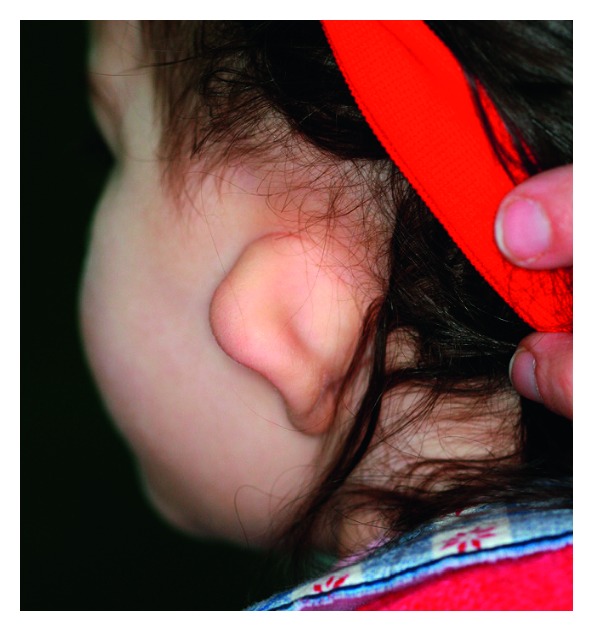
Left external ear malformed, with a rudimentary auricle.

Facial examination showed an asymmetry with the typical three-dimensional deformity on the affected side: on the sagittal dimension, the chin was deviated from the midline; on the vertical plane, the mandibular border was cranially displaced; and on the transversal plane, the face resulted less expanded (Figure 1). The left auricle was hypoplastic (Figure 2).

Both condyles showed appropriate movements, and the maximum mouth opening was at normal range.

Computer tomography (CT) scan revealed a hypoplastic condyle and a deficiency in the ramus height on the affected side.

Intraoral examination highlighted the typical canting of the occlusal plane, as the reduction in ramus length created the condition for a vertical maxillary growth deficiency on the affected side.

Based on clinical and radiographic findings, a diagnosis of grade IIa HM according to Kaban classification was made.

Objectives of treatment planning were (1) to improve mandibular growth in the sagittal and vertical dimension, (2) to free the vertical maxillary growth on the affected side and to correct the occlusal plane canting, and (3) to improve the overall facial symmetry.

It was decided to delay a mandibular osteodistraction and to try a functional approach using an asymmetrical functional activator (AFA).

## 3. Description of the Appliance Design

**Figure 3:**
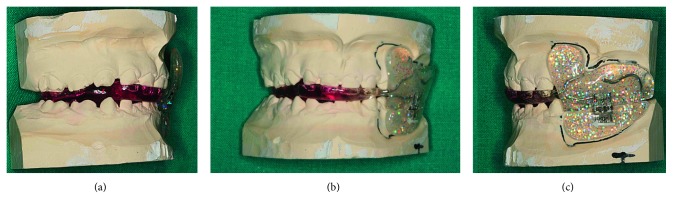
Asymmetrical functional activator (AFA): (a) and (c) lateral views; (b) frontal view.

**Figure 4:**
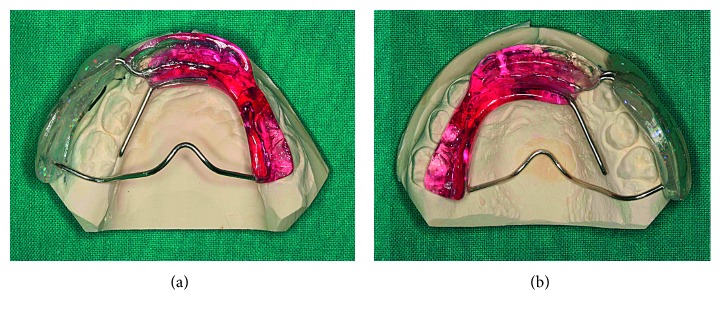
Asymmetrical functional activator (AFA): (a) lower occlusal view; (b) upper occlusal view.

The AFA has a “hybrid” design, being a combination of the two following functional appliances: biteblock components of the bionator and the vestibular shields of the Frankel appliance on the affected side (Figures 3 and 4).

On the affected side, it is necessary to free the vertical growth of the maxilla, maintaining upper and lower teeth apart; thus, the ideal appliance is the Frankel I function regulator. This device maintains the vertical dimension by the means of the buccal shields, avoiding any occlusal contact. Allowing the passive vertical eruption of the upper teeth, the appliance corrects the occlusal plane canting. The soft tissue tension due to the buccal shields improves the stretching and lengthening of the soft tissues too. Buccal shields are supplied with a screw, which is progressively activated in order to increase the vertical dimension.

On the healthy side, an Andresen functional appliance is indicated both to avoid dental eruption and to guide the mandible in the therapeutical position improving the chin symmetry.

The acrylic and the wire elements contribute to create the correct setting for skeletal and dentoalveolar correction by forcing the mandible in the correct position in the three dimensions of the space.

## 4. Treatment Procedure and Results

An alginate impression was taken, and a construction wax bite was registered with the mandible in advanced and symmetric position in order to bring the chin to the midline and to correct the vertical height deficiency on the affected side.

**Figure 5:**
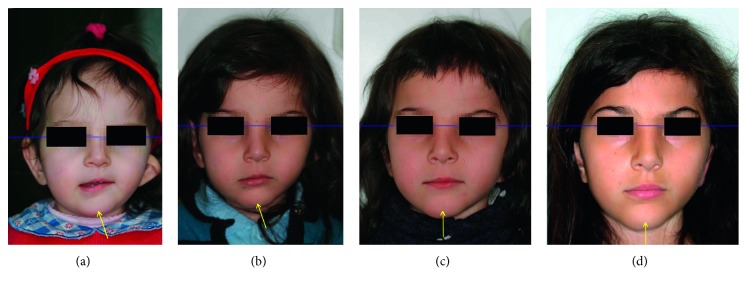
Frontal photographs showing changes in position of the chin point during treatment and follow-up: (a) 2 years and 1 month; (b) 3 years and 11 months; (c) 4 years and 10 months; (d) 7 years and 8 months.

The functional treatment with AFA began at the age of 4, and it was performed for 30 months with good compliance and nearly full-time wearing of the appliance. After this period, the patient showed a favourable response. Particularly, maxilla-mandibular growth on the malformed side developed sufficiently to reestablish normal occlusion and an almost complete facial symmetry, with levelling of the occlusal plane and oral commissure (Figures 1 and 5).

The treatment continued for another 6-month period with the patient wearing the appliance only by night. At the age of 7, the active therapy was interrupted, and the patient underwent regular follow-up twice a year in order to monitor facial growth and dental eruption. During this period, no additional orthodontic or surgical procedures were performed.

## 5. Discussion

Therapy of HM is still controversial, particularly with regard to the possibility of modifying skeletal discrepancy and of redirecting growth on the affected side with a functional approach [7, 8].

Successful treatment of facial asymmetry by functional appliances in the young child following early fracture of the mandibular condyle has extensively been reported [9–13]. Similarly, topics concerning the growth of the mandible and therapeutic approaches to influence it have been addressed in numerous clinical and experimental studies [14–17]. Based on these findings, patients with a recognizable condylar deformity, such as those in type I and IIa HM, might benefit from functional stimulation influencing condylar growth [18–20].

On the other hand, according to few authors, asymmetry tends to recur after orthopaedic treatment in subjects affected by true HM form, which may later require orthognathic surgical correction of skeletal deficiencies [21–23]. Conversely, long-term satisfactory results are more likely to be observed in abnormalities due to trauma, infections, or surgical iatrogenic deformities; these conditions are frequently misdiagnosed as HM, even though they are limited to the bony structure of the mandible without involvement of the soft tissue and neuromuscular pattern [24, 25].

The present case demonstrated a good outcome of functional appliance therapy in a growing patient with type IIa HM. Particularly, the use of the AFA seemed to induce a complete and balanced maxillary-mandibular growth and to be effective in terms of improvement of chin symmetry, occlusal plane, and oral commissure levelling.

Management of the occlusal canting is usually required both in orthodontic camouflage and presurgical orthodontic treatment of patients with HM: early orthopaedic intervention is focused on the differential control of teeth eruption between the opposing arches and the prevention or correction of dentoalveolar adaptation to the asymmetry; by contrast, after growth, the levelling of occlusal plane might require fixed appliances combined with the use of miniscrews [26–28].

Conceivably, the reported favourable result is related to the special conditions of the present case. In fact, the very early age, the excellent cooperation, and the timing of treatment may affect the success of the treatment of asymmetric facial growth [29, 30]. Early interceptive orthodontic treatment is frequently recommended both in children with craniofacial anomalies and in nonsyndromic patients for the positive effect on skeletal growth and dental development [31–34]; moreover, in growing subjects, it may improve nasal function and prevent the development of respiratory disorders [35–37] and of muscle function disturbances [38].

The long-term stability of facial aesthetic is demonstrated by photography, which is the standard means of documenting facial appearance [39]. However, three-dimensional (3D) techniques should be considered the ideal tool for assessment of craniofacial morphology in patients with skeletal asymmetry: compared to conventional two-dimensional representation, 3D evaluation overcomes problems in head positioning and may be used to quantify hard/soft tissue deficiency, as well as to monitor and measure the effects of both growth and treatment [40, 41]. Particularly, objective measurements of the skeletal response can be performed on pre- and posttreatment 3D computerised tomography (CT), documenting incremental growth of the mandible. Given the mild form of the present case, we chose not to perform such exam during the follow-up. Interestingly, noninvasive methods, such as laser surface scanner, stereophotogrammetry, or ultrasonographic measurements, are being intensively investigated in order to make a quantification of facial topography without ionising radiation [42–47].

As for other craniofacial malformations, the role of orthodontists is very important in the interdisciplinary management of patients with HM, especially in cases with mild mandibular and soft tissue deficiencies [48–52]. Problems related to facial appearance may cause severe psychosocial disturbances [53]; thus, improvement of patient aesthetic appearance is also part of treatment objectives [54].

Proper orthopaedic intervention in the early age may induce an improvement of aesthetics, function, and psychological aspects, reducing or even eliminating the need for maxillary and mandibular osteotomies in late adolescence.
